# Recreation Bureau Notes

**Published:** 1893-06

**Authors:** 


### RECREATION BUREAU NOTES.

The complete transformation of the famous Sterlingworth Inn. Lakewood, New York, into a Sanitarium, is a notable one

Where formerly only those on pleasure bent, sought recreation at the charming resort during the heated term a new class of visitors is rapidly filling the large and elegantly furnished Sterlingworth Inn and Sanitarium, although many of those who have enjoyed its hospitality for a summer are among those who are being benefited by rest and treatment beside the shores of beautiful Chautauqua Lake.

Already open for the season is the Temigewassit House at Plymouth, N. H., and the proprietors, C. M. Morse & Son, expect a successful season.

Old Nantucket is familiarly known the world over. The Springfield conducted by Charles H. Mowry, is one of the best hostelries in the east. Beautiful Circulars of the famous hotel can be had of us if desired.

There is no more popular hotel at Saratoga than the United States, its popularity is world wide

The Thousand Islands has been truly pictured as the prettiest spot on the globe and along its shores are manypop ular hostelries. The Westminster is the hotel for the people beautifully located at Westminster Park; its attractive features are well known to those who have visited that wonderful region. Mr. Inglehardt has gotten up beautiful souvenirs of his hotel and region which can be had by addressing our Recreation Bureau or him personally. The Columbia located at Thousand Island Park is about to enter up on its third year and the Journal bespeaks larger success than has heretofore attended it on account of its splendid management. Its cuisine is unequalled.

If you are going to Saratoga write the manager of the Mansion House for special terms. A beautiful hotel, superbly located.

Any reader of the Journal seeking health, with recreation, one of the best Sanitariums in this section is the one located at the Delaware Water Gap. Write us for Circulars gladly sent on application.

The Blue Ridge Springs, Va. is receiving more than usual attention from tourists for the past few years. The Hotel conducted by Phil. F. Brown ranks among the best in the south.

The Providence and Stonington steamship line has commenced its usual summer time-table and is the most popular service out of the metropolis to Boston and other Eastern points.

The New England Conservatory of Music, the best of its kind in this country, invites the many readers of the Journal which at the seashore or mountain side, to send to us for their new handsome circulars giving full details of its workings.

Among our new advertisers we desire to call attention to the offer of Hall's Catarrh Cure. This ailment, so prevalent as it is the East, has surely found a remedy. The Australian Pill is considered superior to any remedy for its special medicinal qualites. Accept of the offer made especially to the Journal.

Recently there have been many Baby Powders thrown upon the market. Inspection has found that they have proved wanting in meeting the desire of mothers. Dr. Fehr's is standard and is recognized as such the world over.

The cuisine departments of the many hotels in this number of the Journal cannot be complete without the Royal Baking Powder which is absolutely pure and recognized as such by chemists of national reputation. Be not deceived. Royal is standard.

Buffalo Lithia Water has the proper medicinal qualities for dyspeptics especially. The springs open June 1st.

This is the season of the year when every one needs a tonic of some kind. Horsford's Acid Phosphate is one of the best offered.

The house keepers of the land know by practiced experience, that when they purchase Van Houten's Cocoa they have the best and goes fatherest.

